# Studying Seabird Diet through Genetic Analysis of Faeces: A Case Study on Macaroni Penguins (*Eudyptes chrysolophus*)

**DOI:** 10.1371/journal.pone.0000831

**Published:** 2007-09-05

**Authors:** Bruce E. Deagle, Nick J. Gales, Karen Evans, Simon N. Jarman, Sarah Robinson, Rowan Trebilco, Mark A. Hindell

**Affiliations:** 1 School of Zoology, University of Tasmania, Hobart, Tasmania, Australia; 2 Australian Government Antarctic Division, Channel Highway, Kingston, Tasmania, Australia; University of Sydney, Australia

## Abstract

**Background:**

Determination of seabird diet usually relies on the analysis of stomach-content remains obtained through stomach flushing; this technique is both invasive and logistically difficult. We evaluate the usefulness of DNA-based faecal analysis in a dietary study on chick-rearing macaroni penguins (*Eudyptes chrysolophus*) at Heard Island. Conventional stomach-content data was also collected, allowing comparison of the approaches.

**Methodology/Principal Findings:**

Prey-specific PCR tests were used to detect dietary DNA in faecal samples and amplified prey DNA was cloned and sequenced. Of the 88 faecal samples collected, 39 contained detectable DNA from one or more of the prey groups targeted with PCR tests. Euphausiid DNA was most commonly detected in the early (guard) stage of chick-rearing, and detection of DNA from the myctophid fish *Krefftichthys anderssoni* and amphipods became more common in samples collected in the later (crèche) stage. These trends followed those observed in the penguins' stomach contents. In euphausiid-specific clone libraries the proportion of sequences from the two dominant euphausiid prey species (*Euphausia vallentini* and *Thysanoessa macrura*) changed over the sampling period; again, this reflected the trend in the stomach content data. Analysis of prey sequences in universal clone libraries revealed a higher diversity of fish prey than identified in the stomachs, but non-fish prey were not well represented.

**Conclusions/Significance:**

The present study is one of the first to examine the full breadth of a predator's diet using DNA-based faecal analysis. We discuss methodological difficulties encountered and suggest possible refinements. Overall, the ability of the DNA-based approach to detect temporal variation in the diet of macaroni penguins indicates this non-invasive method will be generally useful for monitoring population-level dietary trends in seabirds.

## Introduction

Information on predator-prey interactions is essential for understanding everything from animal behaviour and population dynamics to the direct and collateral impacts that humans have on ecosystems. In the marine environment, seabirds are important high-order predators, with an annual consumption that matches the biomass extracted by all human fisheries combined [1]. The conventional method for determining prey species consumed by seabirds is through the examination of remains present in their stomachs. Historically stomach samples were obtained through lethal sampling [e.g. 2], [3]; however, during the last 20 years most studies have used stomach flushing as a non-lethal alternative for obtaining these samples [e.g. 4], [5], [6]. While stomach flushing is certainly an improvement ethically, the procedure requires animal capture and can have adverse effects on the sampled birds and/or their offspring [e.g. 7]. This means that the number of stomach samples that can be obtained during a study is often limited due to ethical concerns and that the approach may not be appropriate for studies of threatened species. Sample collection is also often constrained by the operational difficulty of the flushing procedure and is usually restricted to the subset of breeding birds that are feeding their chicks, as these are the only individuals that reliably bring food back to the colony in their stomaches [8]. Dietary studies based on stomach content analysis can be further hindered by a large number of unidentifiable remains in the stomach [9] and recovery biases caused by differential digestion and/or retention of prey remains [10].

Stable isotope analysis of tissue or feathers has also been used to study diet. This approach provides information on trophic position of predators over relatively long periods of feeding. Isotope analysis has been useful in seabird diet studies for assessment of broad dietary shifts and changes in foraging location [e.g. 11], [12], but it cannot provide the fine-scale diet data often sought in food-web studies.

Prey remains in faeces of predators can provide another important source of dietary information. In marine mammals, collection of faeces and identification of hard parts has allowed large numbers of dietary samples to be analysed in population-scale surveys [e.g. 13], [14]. This approach has not been used in studies of seabird diet because very few hard parts are present in avian faeces [15]. The recent development of DNA-based methods to study diet [16] may provide an opportunity to retrieve dietary information from seabird faeces since the methodology does not rely on visually-identifiable prey remains surviving digestion [16], [17]. The majority of genetic diet studies carried out on vertebrates have focused on identification of prey tissue remains recovered from stomach contents [e.g. 9], [18] or hard parts in faeces [19], [20]. The approach has also been applied to more highly processed amorphous faecal material in some instances [e.g. 17], [21], [22]–[24]. Two previous studies that examined prey DNA in dietary samples collected from a suite of marine predators have shown it is possible to recover prey DNA from faeces of Adélie penguins (*Pygoscelis adeliae*) [17], [22]. However, only a few faecal samples were analysed and each study focused on detection of a single prey species, therefore many basic questions remain unanswered. Before the approach can be more widely applied to study the diet of seabirds and other vertebrate predators it needs to be evaluated in larger-scale field-based studies targeting a broader array of prey. It would also be useful to compare genetic dietary data from faeces with information obtained through conventional dietary analysis in order to examine the strengths and weaknesses of each method. Another issue which needs to be addressed is whether a DNA-based approach can provide ecological data on a scale suitable for detecting fine-scale temporal or spatial variation in diet.

Here, we examine the diet of macaroni penguins (*Eudyptes chrysolophus*) at Heard Island during the chick-rearing phase of their annual cycle. Macaroni penguins have an estimated population size of more than 11 million breeding pairs throughout their sub-Antarctic distribution, making them the most numerous penguin species [25]. The diet of this penguin has been the subject of several studies due to the importance of this species in the Southern Ocean ecosystem [1] and because there have been substantial decreases in population size at many of their breeding sites over the last three decades [e.g. 26]. Dietary studies using conventional stomach content analysis have been carried out in colonies at South Georgia [26], [27], Marion Island [28], [29] and Heard Island [4], [30]. At Heard Island over a million pairs of macaroni penguins rely on food obtained from the surrounding ocean to raise chicks each year [31]. Information available on their diet indicates they consume primarily euphausiids, myctophid fish and, to a lesser extent, amphipods [4], [30]. The only study that has examined diet over a chick-rearing period at Heard Island reported a shift in diet from primarily krill to almost exclusively myctophid fish during this period [4]. Dietary changes are commonly reported in penguins during chick-rearing [32], [33]. Knowledge of such changes is necessary for understanding and managing the ecosystem in which the penguins are foraging.

Stomach content and faecal samples were collected from macaroni penguins on Heard Island during the 2003/2004 breeding season. We obtained dietary data from these samples by performing a conventional stomach content analysis as well as a molecular analysis of prey DNA extracted from faeces. To obtain genetic data from the faecal samples we used two approaches. First, we determined the presence or absence of DNA from five potential diet items by applying PCR tests that specifically amplify DNA from targeted groups of prey. Second, DNA was amplified from faecal samples using primers conserved in prey groups and this DNA was cloned and sequenced to determine its identity. The specific objectives of the study were: (1) to investigate the ability to retrieve data on penguin diet through DNA-based analysis of faeces collected in the field; (2) to compare the dietary information obtained by conventional and DNA-based approaches; and (3) to determine if previously reported intra-seasonal shifts in Heard Island macaroni penguin diet are recurring and if so, whether these trends can be detected using DNA-based methods. The data we present on the diet of macaroni penguins at Heard Island will also provide information crucial for informed management of this remote World Heritage listed area.

## Materials and Methods

### Study site, sample collection and DNA extraction

The macaroni penguin diet samples were collected between December 20^th^ 2003 and February 16^th^ 2004 at the Capsize Beach breeding colony on Heard Island. This remote uninhabited island is located in the Indian Ocean sector of the Southern Ocean (53°05′S, 73°30′E). The waters around the island support trawl and bottom-longline fisheries and also include a 65000 square kilometre highly protected marine reserve (see www.heardisland.aq/). The breeding cycle of macaroni penguins is synchronised within a colony [34], and our sampling period covered the two distinct phases of chick-rearing: (1) guard stage, when females take short foraging trips to provision the young while the male remains at the colony to guard/brood the chick (December 20^th^–January 14^th^); and (2) crèche stage, where both sexes take longer foraging trips and both provision the chick (January 15^th^–February 16^th^).

A total of 69 adult penguins were captured as they returned to the colony and their stomach contents were collected using the water-offloading technique [as described in 5]. The majority of the birds (n = 61) were equipped with externally attached data loggers prior to leaving the colony as part of a concurrent study on foraging behaviour [35]. A maximum of two stomach flushes were performed and individuals were marked to ensure they were sampled only once. The recovered material was drained through a sieve with 0.5 mm mesh size to remove excess water, and then preserved in 70% ethanol. We had planned to obtain faecal samples for genetic analysis from the same birds that were stomach flushed, our permits allowed collection of up to 100 stomach samples along with corresponding faecal samples. However, very few of the birds defecated on capture and faeces therefore had to be collected from non-stomach flushed penguins over the same time period (n = 88). The faecal samples were collected immediately after defecation and stored for approximately one year in 70% ethanol at 4°C. Before DNA extraction, samples were centrifuged for 30 s at 4000×g and the storage ethanol was poured off. DNA was extracted from roughly 100 mg of pelleted material, using the QIAamp DNA Stool Mini Kit (Qiagen) with minor modifications described previously [21]. The DNA was eluted in 100 µL Tris buffer (10 mM). Blank extractions were included in each batch to monitor for cross-over contamination.

### Stomach content analysis

Stomach samples were emptied into a sorting tray and washed in water to settle out fish otoliths and squid beaks. Once these hard parts were removed, the samples were drained on a 0.5 mm sieve, blotted dry and the total mass of the sample was taken. To determine the composition of different prey groups by mass, a 30 g sub-sample was analysed in detail (for samples<30 g the whole stomach sample was analysed). Each sub-sample was examined under a dissecting microscope and divided into five broad prey classes (euphausiids, fish, amphipods, cephalopods or unrecognisable material). The mass of each component was recorded. For the calculation of composition by mass for each sample, the unidentifiable component of the sub-sample was assumed to contain the same proportions of prey as the identifiable component, and the sub-sample was assumed to be representative of the entire sample [3]. The reconstituted mass of the diet is calculated in many diet studies, but was not determined here for two reasons. First, in samples from early foraging trips, the relatively low level of digestion for most samples meant that the composition by mass could be determined directly. Second, many of the samples from foraging trips later in the season were more completely digested. Therefore, the otoliths that had accumulated within these samples likely represent more than one meal and calculation of mass based on these relatively robust structures are unlikely to provide a balanced view of the diet.

To further identify prey present in the diet, otoliths and squid beaks were identified where possible using published keys [36], [37]. To identify the euphausiids, and to determine their species composition by number, up to 100 randomly selected individuals were identified per sample [3], [38]. Amphipods were identified using unpublished reference material from the Australian Government Antarctic Division.

### Genetic analysis of faeces: presence/absence detection

For each faecal sample, the presence/absence of DNA from particular prey was determined with five separate PCR assays using group-specific primers (Table 1). The PCR assays were chosen to detect prey items that were previously identified in the diet of macaroni penguins at Heard Island. The following prey groups, or species, were tested for: (i) euphausiids; (ii) the myctophid fish, *Krefftichthys anderssoni*; (iii) fish from the suborder Nototheniodei; (iv) amphipods; and (v) cephalopods. Two primer sets were specifically developed for use in the current study, EuphMLSU and KaMLSU, targeting euphausiids and *K. anderssoni* respectively. The specificity of these primer pairs were initially evaluated *in silico* using sequences obtained from GenBank and aligned with ClustalX (Table S1). The KaMLSU primer binding site is present in the monospecific species *K. anderssoni*, but is not conserved in other myctophid species. This primer set was tested on genomic DNA from closely related myctophid fish (*Electrona carlsbergi*, *E*. *antarctica*, *K. anderssoni*) and a channichthyid *Champsocephalus gunnari*; as expected only *K. anderssoni* produced PCR products. The EuphMLSUF primer binding site is conserved in the euphausiid genera we were targeting and is not conserved in sequences available from non-euphausiid crustaceans (Table S1). The specificity of these primer sets was also verified through sequencing of amplified products and BLAST analysis (outlined below).

**Table 1:** PCR primers used in the present study.

Target (taxon–gene)	Primer name	Sequence 5′→3′	Product size (bp)	Annealing temperature	Reference
**Euphausiid** a–mitochondrial 16S rDNA	EuphMLSUF	tttattggggcgataaaaat	169	54°C	This study
	EuphMLSUR	tcgaggtcgyaatctttcttgt			This study
***Krefftichthys anderssoni*** a–mitochondrial 16S rDNA	KaMLSUF	cccacatcaaatacccccta	169	55°C	This study
	KaMLSUR	gggtcattggtggtcagaag			This study
**Nototheniodei**–mitochondrial 16S rDNA	NotoMLSUF	ccctatgaagcttyagacrta	∼275	55°C	[22]
	NotoMLSUR	ccttgttgatawggtctctaaaa			[22]
**Amphipoda**–nuclear 18S rDNA	AmphNSSF1	ctgcggttaaaaggctcgtagttgaa	204–375	51°C	[56]
	AmphNSSR1	actgctttragcactctgatttac			
**Cephalopoda**–nuclear 28S rDNA	Squid28SF	cgccgaatcccgtcgcmagtaaamggcttc	∼180	55°C	[21]
	Squid28SR	ccaagcaacccgactctcggatcgaa			[21]
**All prey** a–mitochondrial 16S rDNA	16S1F-degenerate	gacgakaagacccta	180–270	54°C	This study
	16S2R-degenerate	cgctgttatccctadrgtaact			This study

a See text and Table S1 for further details.

PCR amplifications were performed in 25 µL reactions containing 0.4 µM of each primer, 0.2 mM dNTPs, 2.0 mM MgCl_2_, 1× BSA (New England Biolabs),1× AmpliTaq Gold buffer and 0.625 units AmpliTaq Gold (Applied Biosystems). Template was 1 µL of the DNA extract. Thermal cycling conditions were as follows: 94°C for 10 min then 35 cycles (94°C for 30 s/primer-specific annealing temperature for 30 s/72°C for 45 s) followed by 72°C for 2 min. Aerosol-resistant pipette tips were used with all PCR solutions and negative control reactions (extraction control and a distilled water blank) were performed with each set of PCR amplifications. PCR products were separated by electrophoresis in 1.8% agarose gels and visualised by staining with ethidium bromide.

### Genetic analysis of faeces: clone library analysis

PCR clone libraries were produced from representative faecal samples which contained prey DNA and clones from these libraries were sequenced. Two primer sets were used to produce clone libraries:

1. The first primer set was considered to be universal for prey DNA since it targets a primer binding region of the mitochondrial 16S rDNA gene that is highly conserved in fish, cephalopods and crustaceans [16S1F and 16S2R-degenerate; based on primers described in 39]. The adenosine nucleotide on the 3′ end of the forward primer does not match the primer binding region in birds, but is conserved in the target prey groups (Table S1). This single nucleotide mismatch was incorporated into the primer to prevent the amplification of penguin DNA. Using this primer set we amplified DNA from ten faecal samples and cloned the products. Six sequences were obtained from each sample, giving a total of 60 sequences from these libraries.
2. The second primer set was the euphausiid primer pair described above (Table 1). With this primer set we amplified DNA from ten faecal samples and ten sequences were obtained from each sample, giving 100 sequences from these libraries.

PCR amplifications were carried out following the protocol outlined in the previous section. Products were cloned using the TOPO TA cloning system following instructions of the manufacturer (Invitrogen). Colonies containing recombinant clones were cultured and plasmid DNA was purified by alkaline lysis [40]. Sequencing was carried out on 300 ng of plasmid DNA using the BigDye Terminator Version 3.1 cycle sequencing reagents (ABI). Capillary separation was performed on AB3730xl sequencing platforms at the Australian Genome Research Facility. Chromatograms were examined by eye to check base calling in the program Chromas2 (Technelysium).

### Data analysis

To compare diet composition between the two stages of chick-rearing (guard and crèche) we applied the non-parametric method, ANOSIM (analysis of similarity) to both the stomach content data and the faecal genetic data, using PRIMER statistical software (version 5.2.9). The procedures used followed those outlined in [41]. For the stomach content data, the mass of prey groups present within each sample was converted to percentage composition. These data were used in preference to frequency of occurrence data since the incorporation of mass measurements provides a more accurate view of diet (by preventing prey taken in small quantities being over-represented). For the genetic results, no weighting of the detection results were possible, so comparisons were carried out using the presence/absence detection data. For each dataset a similarity matrix was generated using the Bray–Curtis similarity measure. ANOSIM tests were run on the matrices using 9999 permutations to test for statistically significant differences in diet composition between samples collected during guard and crèche stage. The contribution of each prey category to the average dissimilarity between the chick-rearing stages was calculated using the similarity percentages procedure (SIMPER) in PRIMER.

To identify sequences obtained from our clone library analysis, sequences were aligned and grouped into clusters of nearly identical sequences (i.e. sequences differing by one or two base substitutions). All unique sequences were compared with sequences in GenBank using the BLAST program [42]. Matches that were identical with sequences present in GenBank over the entire fragment and that were different from other species within the same genera were considered to provide species level identification. If all sequences within a nearly identical cluster shared a common closest match they were considered to be the same species. If no matches of 100% were present in the database for any members of a nearly identical cluster, the consensus sequence was classified to the lowest taxonomic level possible with reference to the closest BLAST matches. To aid in the identification of sequences from euphausiids we generated sequence data from reference specimens of *Thysanoessa macrura, Euphausia frigida, E. tricantha* and *E. vallentini* (GenBank accession numbers: DQ356238, DQ356239, DQ356240 and DQ356241). This was accomplished by extracting DNA from muscle tissue and directly sequencing PCR products generated using the primers 16Sar-5′ and 16Sa-3′ [43]. These conserved primers amplify the 3′ region of the mitochondrial 16S rDNA gene and encompass the sequence amplified by our euphausiid primer pair.

## Results

### Stomach content analysis

Of the 69 stomachs sampled, 11 were empty and an additional five were excluded from further analysis because they had a mass of less than 5 g. The remaining 53 stomach samples had a mean mass of 75 g (s.d. = 51 g, range = 12–216 g); these included 35 collected during guard stage and 18 collected during crèche stage of chick-rearing (data for individual samples given in Table S2). Overall, euphausiids formed the largest component of the stomach samples by mass (69%). Fish ranked second (22%), followed by amphipods (8%) and then cephalopods (<1%); (Table 2). The same ranking of relative importance of these prey groups was obtained based on the frequency of occurrence data, although the importance of prey items present in small amounts in some samples was exaggerated (e.g. cephalopods and euphausiids during crèche stage) (Table 3). ANOSIM tests detected significant differences in the prey mass proportions during guard and crèche stages (global R of 0.317; p<0.01). This difference was due to an increase in the amount of fish and amphipods, with a corresponding decrease in the importance of euphausiids during the later stages of chick-rearing (Table 2). SIMPER analysis show a percentage dissimilarity of 54% between stages, and the contribution of the prey categories to the discrimination were: euphausiids (46%), fish (32%) and amphipod (22%).

**Table 2:** Stomach sample composition of the main prey groups consumed by macaroni penguins during chick-rearing (based on total wet mass of prey components in all samples combined).

	Total (n = 53) a		Guard (n = 35)		Crèche (n = 18)	
	(g)	(%)	(g)	(%)	(g)	(%)
Euphausiids	2760.3	69	2169.7	83	590.6	43
Fish	884.2	22	424.5	16	459.7	33
Amphipods	327.4	8	6.8	<1	320.6	23
Cephalopods	10.9	<1	1.0	<1	9.9	1
Total	3982.8	100	2602.0	100	1380.8	100

a Data on the mass and composition of stomach contents from individual birds is given in Table S2

**Table 3:** Comparison of percent frequency of occurrence data (% FO) of main prey groups identified through conventional stomach content analysis and presence/absence genetic analysis of faeces.

Prey Item	Stomach data		Faecal DNA data	
	Guard (n = 35)	Crèche (n = 18)	Guard (n = 13)	Crèche (n = 26)
	% FO	% FO	% FO	% FO
Euphausiids	97	100	85	15
*K. anderssoni*	63 a	94 a	31	77
Nototheniodei	6	6	0	23
Amphipods	51	72	15	46
Cephalopods	9	33	0	15

a Based on otolith recovery

The prey species identified in the stomach contents included at least three species of euphausiids, three fish, two amphipods, one squid and a chaetognath (Table 4). Two species of krill (*E. vallentini* and *T. macrura*) made up the vast majority of the identified euphausiids. Both of these species have closely related sister taxa which occur in the vicinity of Heard Island (*E. frigida* and *T. vicina*) and the fragile taxonomic features distinguishing these species were missing from many of the partially digested samples, therefore the occurrence of these sister taxa in the samples could not be discounted. Another species of euphausiid, *E. tricantha*, was found in very small numbers (only four specimens out of more than 3000 euphausiids identified). *E. vallentini* was the dominant species consumed during the early part of the study and it was almost completely replaced by *T. macrura* in samples collected during the latter part of the study. Of the 3355 fish otoliths recovered from the stomachs, 3255 were from the myctophid *K. anderssoni*, a single otolith was from *Electrona antarctica* and the remaining 99 could only be identified as from the family Myctophidae. One intact fish was recovered and identified as *Channichthys rhinoceratus*; four additional digested channichthyid icefish were recovered but could not be further identified, and several small unidentifiable fish were also present in the samples. Almost all identified amphipods belonged to a single species, *Themisto gaudichaudii*; the only exception was a single specimen identified as *Hyperia macrocephala*. From the squid remains present in the samples only one lower beak was large enough to allow identification and this beak came from the squid *Galiteuthis glacialis*. Chaetognaths (n = 5; *Sagitta* sp.) were present in one stomach sample, and represented less than 0.05% of the total mass of the diet samples.

**Table 4:** Comparison of prey identified by conventional stomach content and faecal DNA analysis.

Prey Group	Species ID	Stomach contents	Faecal DNA presence/absence	Faecal DNA clone libraries	# of clones–library	GenBank accession	% similarity of match
Euphausiids		+	+	+			
	*Thysanoessa macrura*	+		+	70–**Euphausiid**	DQ356238	100%
	*Euphausia vallentini*	+		+	28–**Euphausiid**	DQ356241	100%
	*Euphausia frigida*			+	2–**Euphausiid**	DQ356239	100%
	*Euphausia tricantha*	+					
Fish		+	+	+			
	*Krefftichthys anderssoni*	+		+	42–**Universal prey**	AB042176	100%
	*Electrona antarctica*	+		+	2–**Universal prey**	AY141397	99%
	*Champsocephalus gunnari* a			+	4**–Universal prey**	AY249471	100%
	*Harpagifer* sp.^b^			+	1**–Universal prey**	AY520130	100%
	Nototheniinae sp.^c^			+	4**–Universal prey**	DQ356243	99%
	*Channichthys rhinoceratus*	+					
	*Unidentified Acanthopterygii*			+	6**–Universal prey**	DQ356242	82%
Amphipods		+	+				
	*Themisto gaudichaudii*	+					
	*Hyperia macrocephala*	+					
Cephalopods		+	+	+			
	*Gonatus antarcticus*			+	1**–Universal prey**	AY681032	100%
	*Galiteuthis glacialis*	+					
Chaetognatha							
	*Sagitta sp.*	+					

Genetic results from presence/absence PCR tests and from sequence data obtained through the analysis of clone libraries are shown.

a sequence is also 100% match with *C. esox,* but this species not found near Heard Island and is the sole congener

b sequence is 100% match with *H. kerguelensis* and *H. antarcticus*

c sequence is 98–99% match with *Gobionotothen* spp. and *Notothenia coriiceps*, both are within the sub-family Nototheniidae

### Genetic presence/absence detection in faecal samples

**Figure 1:**
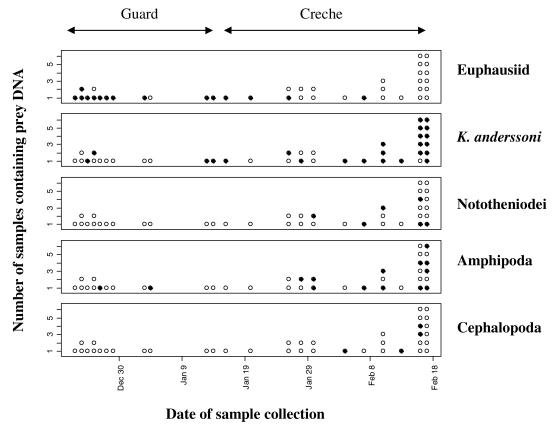
Summary of the detection data from the five PCR tests carried out on each faecal sample. Boxes represent results from PCR tests designed to detect prey groups labeled on right. Each dot represents a faecal sample which tested positive for at least one prey item (39 in total); a filled dot indicates detection of the particular prey group. The horizontal axis shows the date the samples were collected.

Slightly less than half of the faecal samples (39 out of 88) tested positive for one or more of the prey groups targeted with PCR tests (Figure S1). In these 39 informative samples, euphausiid DNA was detected in 15 samples (38%), *K. anderssoni* DNA in 24 samples (62%), Nototheniodei DNA in 6 samples (15%), amphipod DNA in 14 samples (36%) and cephalopod DNA in 4 samples (10%); (Fig. 1). In these data there was a significant difference in the prey items detected between the guard and crèche stage (ANOSIM global R of 0.346; p<0.01). As with the stomach mass data, euphausiids were more prevalent in guard stage samples compared with crèche stage samples and the opposite was true for fish and amphipods (Table 3). The contribution of these prey categories to the percentage dissimilarity between stages (73%; SIMPER analysis) were: euphausiids (36%), *K. anderssoni* (30%) and amphipod (20%).

### Genetic clone library analysis of faecal samples

In the 60 sequences obtained from clones produced using the universal prey primer set (16S F+R degenerate) no sequences from the predator were obtained. This indicates the single nucleotide polymorphism differentiating the primer binding site of macaroni penguins from their prey was effective in suppressing amplification of predator DNA (Table S1). Seven distinct prey DNA sequences were recovered (Table 4 and Table S3). These sequences were almost entirely derived from fish, with the majority of sequences matching the myctophid fish *K. andersoni*. Other fish represented in the clone libraries include another myctophid (*E. antarctica*), three species from the suborder Nototheniodei (*Champsocephalus gunnari*, Harpagifer sp. and a fish belonging the sub-family Nototheniidae) and one fish species whose sequence does not closely match any of the species represented in GenBank. The only non-fish prey detected was from a single sequence identified as the squid *Gonatus antarcticus*.

**Figure 2:**
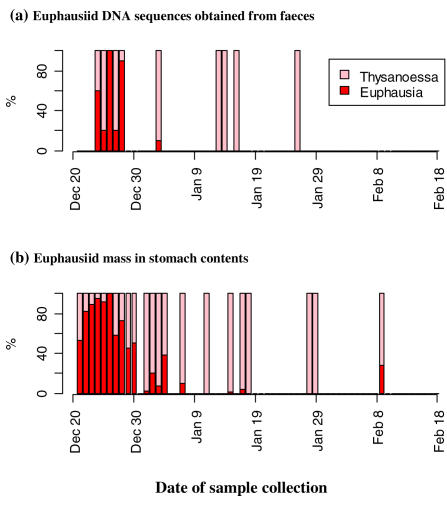
Proportional breakdown of two euphausiid genera in diet samples collected over the sampling period. (a) Based on 100 sequences obtained from cloned PCR products amplified using a euphausiid-specific primer set (ten sequences from each of ten clone libraries); (b) Based on numbers present in stomach samples (data from multiple stomach samples collected on the same day were pooled).

In the clone libraries produced from PCR amplifications using the euphausiid primer set, three species of krill were identified in the 100 clones sequenced. Seventy of the clones matched *T. macrura*, 28 matched *E. vallentini* and 2 matched *E. frigida* (Table 4 and Table S3). We classified the *T. macrura* sequences based on a 100% match but there is no sequence data available for the closely related species *T. vicina*. As a result, we cannot discount the possibility that this species was present but could not be distinguished from *T. macrura*. The proportion of clones from the two euphuasiids changed over the sampling period, with *Euphausia* sp. dominating the krill component of the diet in samples collected during the early part of the sampling period and *T. macrura* identified exclusively in samples collected later. This follows the trend seen in the stomach content data (Fig. 2).

## Discussion

The stomach content data we collected present a picture of macaroni penguin diet that is generally consistent with the results obtained in two previous diet studies carried out at Heard Island [4], [30]. In all three studies the majority of the diet was composed of a combination of two euphausiids, *E. vallentini* and/or *T. macrura*, and one species of myctophid fish, *K. anderssoni*. The amphipod *T. gaudichaudii* was also taken in significant numbers by some penguins sampled in each study. Squid was a very minor component of the diet in all studies. We found that the dominant species of euphausiid in the diet shifted from *E. vallentini* to *T. macrura* over the course of our study; this has not been observed previously. The euphausiids identified by Green *et al.* [4] in stomach contents collected during chick-rearing were almost exclusively *E. vallentini*. Klages *et al*. [30] reported that both *euphausiid* species were consumed. Shifts in the principal crustacean species being eaten have been observed previously in macaroni penguins at Marion Island [28], and these are likely due to temporal changes in prey availability [see discussion in 4]. We also found a reduction in the reliance on euphausiids during later stages of chick-rearing. This was similar to that observed by Green *et al*. [4], the only other Heard Island study encompassing both guard and crèche stages. In our study, euphausiids constituted 83% of the diet by mass during guard stage and 43% by mass during crèche stage, the difference being made up by an increase in the amount of fish and amphipods. This dietary change possibly results from a change in location of foraging area utilised during crèche; at this stage of chick-rearing longer foraging trips are possible as guarding/brooding is no longer required and both parents can provision the chick [32], [44].

The DNA-based faecal analysis provided some promising results. Using group-specific PCR assays we were able to detect DNA from a range of pre-defined prey groups in the faecal samples. Even with the relatively small number of samples we were able to analyse, the dietary shifts observed in the stomach content mass composition data were apparent in the genetic data from each of the prey groups: euphausiid DNA was more commonly detected in samples collected during guard stage, and DNA from *K. anderssoni* and amphipods increased in prevalence during crèche stage. When frequency of occurrence data is used in conventional stomach content studies it is often inaccurate since prey taken in small quantities are given the same weight as those making up a large proportion of the sample [7]. Our results indicate genetic data from faeces may be preferable to stomach content data when carrying out this level of analysis due to the low diversity of prey within individual faecal samples. If potential prey species are known *a priori* this type of relatively simple DNA detection approach could be effective to monitor trends in prey consumption. It should be noted that comparison of frequency of occurrence data between prey species may be complicated by differences in the sensitivity of prey-specific PCR tests (i.e., if the amount of prey DNA in many samples is close to the sensitivity limits of the PCR tests, then the importance of prey targeted by the least sensitive test will be underestimated).

The cloning/sequencing data provided additional useful dietary information. The change in the dominant euphausiid species being consumed by the penguins during the early part of the study was clearly seen in sequences from the euphausiid clone libraries. This suggests semi-quantitative results could be obtained by this method if amplification efficiency is equal in the targeted prey groups [see discussion below and in 45]. Additional sequence results from the universal prey clone library analysis confirmed the dominance of *K. anderssoni* in the fish component of the diet and also revealed a larger diversity of fish prey in the diet than identified in the stomach content analysis. An increase in the importance of fish from the suborder Nototheniodei was noticeable in the genetic analysis (in both the presence/absence and clone library data). While the otoliths of most Nototheniodei are relatively robust [36], no otoliths from this group were recovered from the stomach contents. One explanation for this could be that very small Nototheniodei were consumed and their tiny otoliths were not recovered. This suggestion is supported by the small size of the few digested Nototheniodei fish that were recovered and by previous reports of unidentifiable Nototheniodei fish larvae in macaroni penguin stomach contents [30]. Another possibility is that the increased detection of these fish in the faecal samples could have resulted from real differences in the diet of the birds that were stomach sampled versus those whose faeces were collected. The stomach samples were obtained from breeding birds (i.e. carefully selected birds with protrusive brood pouches) that were fitted with data loggers as part of a concurrent study on foraging behaviour [35]. In contrast, the faecal samples were randomly collected from penguins present on the beach near the colony (potentially including some non-breeding birds) and these penguins were not carrying data loggers. Both breeding status and instrument attachment could influence the diet of these groups [44], [46].

We did encounter several difficulties in the genetic analysis that could be remedied in future studies. First, a large number of samples contained no amplifiable prey DNA, resulting in a smaller than anticipated sample size. There are several possible reasons for this: samples may have contained PCR inhibitors, DNA may have degraded during storage, or there may not have been any prey DNA present because the defecating bird had not fed recently. The last explanation is almost certainly true in some cases since nearly 20% of the birds that were stomach flushed had empty stomachs. It would be useful to examine faeces collected from captive birds to determine prey detection rates and the persistence of a detectable genetic signal after prey ingestion [21]; this might clarify the reasons for the high incidence of negative results. Regardless of the reason, it is prudent to collect large numbers of samples to compensate for the proportion of samples that do not contain any amplifiable prey DNA. In the case of penguins at accessible colonies, samples are easily obtained and the initial PCR screening of samples for prey DNA can be done relatively quickly and cheaply.

Another technical difficulty was that the clone libraries generated using the universal prey 16S primers (degenerate primers specifically designed to amplify DNA from a wide variety of prey) did not represent the diversity of prey in the penguins' diet–almost all the sequences obtained in this analysis came from fish. Two prey groups, euphausiids and amphipods, were conspicuously absent. It has previously been shown that prey DNA proportions in faeces may provide a somewhat biased reflection of proportions of prey ingested, due to differences in prey DNA density and DNA survival during digestion [45]. However, the cause of the major bias found in the present study is likely methodological, resulting from differences in primer binding efficiency for the different prey groups targeted. The degenerate primer set used to create the universal prey libraries was capable of amplifying DNA from fish, euphausiids and squid (determined in preliminary testing), but the melting temperatures of primers matching the euphausiid binding sites are lower than those of the corresponding fish primers (Table S4). This could cause differential PCR amplification of the mixed template present in the faecal samples [47]. With regard to the lack of representation of amphipods, the primers were designed with reference to amphipod sequences from the gammaroidean suborder that were available in GenBank. Testing carried out at the end of the study revealed that PCR products could not be amplified from genomic DNA of the hyperiidean species, *T. gaudichaudii*, with this primer set. To avoid biased conclusions in future studies that use a universal primer approach, an increased emphasis should be placed on primer selection. In an ideal situation, completely conserved primer binding sites could be targeted so DNA from all prey targeted would be amplified simultaneously, with equal efficiency. Unfortunately very few primers meet this criterion and also amplify short, informative DNA regions that are well represented in GenBank. One way to safeguard against spurious results caused from primer-specific bias would be to analyse clone libraries produced from multiple primer sets to allow for cross-validation [48].

It might be assumed that the identification of sequences obtained in faecal analyses would be quite limited due to the short length of DNA that can be amplified [49]. However, in the current study the taxonomic resolution obtainable in some groups was very good. Using mitochondrial 16S sequences isolated from faeces it was possible to distinguish between two species in the genus *Euphausia* (*E. vallentini* and *E. frigida*) even though these species were not morphologically distinguishable in the stomach content analysis. In some groups (e.g. notothenioid fish) differentiation between some closely related species was not possible due to lack of variation in the targeted mitochondrial 16S region. Before a study is initiated, *a priori* analysis of genetic variation in potential prey groups could be carried out to determine if the taxonomic resolving power of a particular marker is suitable for the question being addressed [e.g. 50]. The primary limitation we encountered in the identification of DNA sequences resulted from a lack of reference sequence data. One DNA sequence we obtained is distantly related to several ray-finned fish (approximately 20% sequence divergence) and could not be classified further. This was surprising given the relatively good coverage of this group in GenBank. As discussed above, an entire suborder of amphipods is unrepresented by mitochondrial 16S DNA sequences in GenBank. This is likely to be the case for many groups of marine invertebrates, making identification in diet samples possible only with concurrent sequencing efforts of the relevant potential prey taxa. One of the compensatory features of DNA-based identification is that sequence data obtained in different studies is easily catalogued and taxonomic classification of sequences can be improved retrospectively. The growth in available sequence data for some genes, such as the mitochondrial cytochrome oxidase subunit I gene favoured by the international DNA barcoding effort, will be rapid [51]. This protein coding gene is not ideal for the design of non-degenerate primers [52], but it has found some use in diet studies [48], [53]. The continued development of sequence databases that focus on accurate taxonomy and high quality sequence data will facilitate future DNA-based diet studies.

In summary, our stomach content analysis showed that macaroni penguins breeding on Heard Island primarily consume euphausiids during chick-rearing with an increasing reliance on the myctophid *K. anderssoni* and the amphipod *T. gaudichaudii* during crèche stage. The temporal variability of the diet suggests studies encompassing the full breeding season are needed to appreciate the breadth of local resources utilized by penguins. Our study also illustrates that dietary information can be obtained from prey DNA in penguin faeces. Presence/absence PCR tests revealed population-level dietary trends congruent with those seen in parallel stomach content analysis. Group-specific PCR and sequencing, such as carried out with the euphausiid primer set, improved taxonomic resolution of prey identification compared with morphological analysis of stomach contents. The use of universal PCR primers potentially provides a powerful method for determining the diversity of prey consumed; however, results should be interpreted cautiously since differential amplification of DNA can cause major biases. The most significant advantage of genetic faecal analysis is that dietary samples can be collected with virtually no disturbance to the birds. With larger sample sizes, better temporal resolution of dietary changes can be attained. The non-invasive nature of the approach will be especially beneficial in studies determining diet of endangered seabirds and in long-term monitoring studies [e.g. 54], [55]. Broader application and refinement of the DNA-based faecal approach will allow a substantial expansion in the amount of information obtainable in seabird diet studies.

## Supporting Information

Table S1 PCR primers designed in the current study aligned with homologous sequences from representative target and non-target.
(0.08 MB DOC)

Table S2 Stomach content data from each individual macaroni penguin sampled. Information provided includes data on the wet mass of each sample, proportions of various prey groups and number of otoliths recovered.
(0.02 MB XLS)

Table S3 Identity of sequences obtained in clone libraries produced from individual penguin faecal DNA samples.
(0.04 MB DOC)

Table S4 Melting temperatures of the degenerate universal primers (16S1F-degenerate and 16S2R-degenerate) used to create clone libraries from penguin faecal DNA samples.
(0.04 MB DOC)

Figure S1 Collection dates for all 88 penguin faecal samples analysed during the study and sample numbers for the 39 penguin faecal samples containing prey DNA. Sample numbers correspond to clone library results in Table S3 and presence/absence results shown in Figure 1.
(0.05 MB DOC)
