# Supplementary material for: Studying Seabird Diet through Genetic Analysis of Faeces: A Case Study on Macaroni Penguins (Eudyptes chrysolophus)
Source: PLoS One. 2007 Sep 5;2(9):e831. doi: 10.1371/journal.pone.0000831 (PMC1959119; doi:10.1371/journal.pone.0000831)
Supplement: Table S1 — PCR primers designed in the current study aligned with homologous sequences from representative target and non-target. (0.08 MB DOC) [file pone.0000831.s001.doc]

**Table S1** *PCR primers designed in the current study aligned with homologous sequences from representative target and non-target.*

| Family | Genus + species | Accession # | Primer name and sequence alignment (5' → 3')^a^ | | Target ^b^ |
| --- | --- | --- | --- | --- | --- |
|  |  |  | **KaMLSUF** | **KaMLSUR** |  |
|  |  |  | CCCACATCAAATACCCCCTA | GGGTCATTGGTGGTCAGAAG |  |
| Myctophidae (Lanternfishes) | *Krefftichthys anderssoni* | AB042176 | .................... | .................... | Yes* |
| Myctophidae (Lanternfishes) | *Electrona carlsbergi* | AB042175 | ...........C.....TC. | .................... | No* |
| Myctophidae (Lanternfishes) | *Protomyctophum thompsoni* | AB042177 | ...........C.....TC. | .................... | No |
| Myctophidae (Lanternfishes) | *Electrona antarctica* | AY141397 | ........G.CCC.TT..CC | .................... | No* |
| Myctophidae (Lanternfishes) | *Gymnoscopelus nicholsi* | AB042173 | ....TG....GC.T...T.C | ..C..T.............A | No |
| Channichthyidae (Icefishes) | *Channichthys rhinoceratus* | AY249476 | .AA.ATA...T........G | ..A..TA.A--.....A..A | No |
| Channichthyidae (Icefishes) | *Champsocephalus gunnari* | AF145409 | .AA.ATAAC....A...... | ..A..TG.A--.....A..A | No* |
| Nototheniidae (Antarctic cods) | *Notothenia coriiceps* | Z32731 | .AG.ATA....GG......C | ..A..T..A--.....A..A | No |
|  |  |  |  |  |  |
|  |  |  | **EuphMLSUF** | **EuphMLSUR** |  |
|  |  |  | TTTATTGGGGCGATAAAAAT | TCGAGGTCGTAATCTTTCTTGT |  |
| Euphausiidae (Krills) | *Euphausia vallentini* | DQ356241 | .................... | ...................... | Yes* |
| Euphausiidae (Krills) | *Euphausia tricantha* | DQ356240 | .................... | ...................... | Yes* |
| Euphausiidae (Krills) | *Euphausia superba* | Z73805 | .................... | ...................... | Yes* |
| Euphausiidae (Krills) | *Euphausia frigida* | DQ356239 | .................... | ...................... | Yes |
| Euphausiidae (Krills) | *Thysanoessa macrura* | DQ356238 | .................... | .........C............ | Yes* |
| Euphausiidae (Krills) | *Nyctiphanes capensis* | AY574930 | .................... | .........C............ | Yes |
| Palinuridae (Spiny lobsters) | *Jasus edwardsii* | AF337979 | ...GC.......GC.GGGG. | .........C..A.C..GCCT. | No |
| Penaeidae (Penaeid shrimps) | *Penaeus monodon* | AF125383 | .GCG.........CGGG... | ..C.A....C..A.C....... | No |
| Mysidae (Mysids) | *Mesopodopsis wooldridgei* | AJ966899 | ..A........A..T..... | .........C....AA...CA. | No |
| Gammaridae (Amphipods) | *Gammarus locusta* | AY926726 | ....C.......G.GTG.G. | .......T.C..A.A..T..A. | No |
|  |  |  |  |  |  |
|  |  |  | **16S1F-degenerate** | **16S2R-degenerate** |  |
|  |  |  | GACGAKAAGACCCTA | CGCTGTTATCCCTA**DR**GTAACT |  |
| Myctophidae (Lanternfishes) | Krefftichthys anderssoni | AB042176 | .....G......... | ..............GA...... | Yes* |
| Gonatidae (Squids) | Gonatus antarcticus | AY681032 | .....G......... | ..............TG...... | Yes* |
| Euphausiidae (Krills) | Euphausia vallentini | DQ356241 | .....T......... | ..............AA...... | Yes* |
| Gammaridae (Amphipods) | *Gammarus locusta* | AY926726 | .....T......... | .A............GA...... | Yes |
| Spheniscidae (Penguins) | Eudyptes chrysolophus | DQ137157 | .....G........G | .............GGG...G.. | No |
| Spheniscidae (Penguins) | Aptenodytes patagonicus | DQ137148 | .....G........G | .............GGG...G.. | No |
|  |  |  |  |  |  |

^a^ Redundancies use the code: K (GT); R (AG); D (AGT)

^b^ Intended PCR targets listed as Yes; * indicates specificity of primer was tested empirically against genomic DNA template.
