# Supplementary material for: Studying Seabird Diet through Genetic Analysis of Faeces: A Case Study on Macaroni Penguins (Eudyptes chrysolophus)
Source: PLoS One. 2007 Sep 5;2(9):e831. doi: 10.1371/journal.pone.0000831 (PMC1959119; doi:10.1371/journal.pone.0000831)
Supplement: Table S3 — Identity of sequences obtained in clone libraries produced from individual penguin faecal DNA samples. (0.04 MB DOC) [file pone.0000831.s003.doc]

**Table S3** *Results from the penguin faecal DNA clone library analysis. Samples given in bold were analysed with both primers sets.*

(**A**) “Universal” prey primers (16S1F and 16S2R degenerate) (**B**) Euphausiid primer pair

| Clone library | Sample  # | Date | # Clones - Species ID^a^ |
| --- | --- | --- | --- |
| **A1** | **4** | Dec 25 | 6 - *K. anderssoni* |
| **A2** | **5** | Dec 26 | 6 - Acanthopterygii |
| **A3** | **12** | Jan 13 | 6 - *K. anderssoni* |
| **A4** | **14** | Jan 16 | 6 - *K. anderssoni* |
| A5 | 18 | Jan 28 | 6 - *K. anderssoni* |
| A6 | 23 | Feb 7 | 3 - *K. anderssoni*  1 - *Harpagifer* sp.  2 - *C. gunnari* |
| A7 | 24 | Feb 10 | 6 - *K. anderssoni* |
| A8 | 27 | Feb 13 | 5 - *K. anderssoni*  1 - *G. antarcticus* |
| A9 | 31 | Feb 15 | 4 - *K. anderssoni*  2 - *E. antarctica* |
| A10 | 34 | Feb 16 | 1 - *K. anderssoni*  2 - *C. gunnari*  3 - Nototheniinae sp. |

| Clone library | Sample  # | Date | *Euphausia/ Thysanoessa*^b^ |
| --- | --- | --- | --- |
| B1 | 3 | Dec 24 | 6/4 |
| **B2** | **4** | Dec 25 | 2/8 |
| **B3** | **5** | Dec 26 | 10/0 |
| B4 | 7 | Dec 27 | 2/8 |
| B5 | 8 | Dec 28 | 9/1 |
| B6 | 10 | Jan 3 | 1/9 |
| **B7** | **12** | Jan 13 | 0/10 |
| B8 | 13 | Jan 14 | 0/10 |
| **B9** | **14** | Jan 16 | 0/10 |
| B10 | 16 | Jan 26 | 0/10 |

^a^ Taxonomic identity of the six clones sequenced from each library. Further details, including GenBank accession numbers, are in Table 4

^b^ Identity of euphausiid sequences in ten clones sequenced from each library.
