# Supplementary material for: Studying Seabird Diet through Genetic Analysis of Faeces: A Case Study on Macaroni Penguins (Eudyptes chrysolophus)
Source: PLoS One. 2007 Sep 5;2(9):e831. doi: 10.1371/journal.pone.0000831 (PMC1959119; doi:10.1371/journal.pone.0000831)
Supplement: Table S4 — Melting temperatures of the degenerate universal primers (16S1F-degenerate and 16S2R-degenerate) used to create clone libraries from penguin faecal DNA samples. (0.04 MB DOC) [file pone.0000831.s004.doc]

**Table S4** *Melting temperatures of the degenerate primers used to create*

*“universal” clone libraries from penguin faecal samples. The taxa-specific primer binding sites and melting temperatures are shown. Size of amplification products are: Fish ~ 250 bp; Cephalopoda ~ 180 bp; and Euphausiid ~ 200 bp.*

| **Primer sequences** (5' → 3')^a^ | Matching taxa | Melting temperature^b^ |
| --- | --- | --- |
|  |  |  |
| **16S1F-degenerate** |  |  |
| GACGA**K**AAGACCCTA | All | — |
| GACGA**G**AAGACCCTA | Fish | 52.58 |
| GACGA**G**AAGACCCTA | Cephalopoda | 52.58 |
| GACGA**T**AAGACCCTA | Euphausiid | 49.34 |
|  |  |  |
| **16S2R-degenerate** |  |  |
| CGCTGTTATCCCTA**DR**GTAACT | All | — |
| CGCTGTTATCCCTA**GG**GTAACT | Fish | 63.89 |
| CGCTGTTATCCCTA**TG**GTAACT | Cephalopoda | 62.22 |
| CGCTGTTATCCCTA**AA**GTAACT | Euphausiid | 60.42 |
|  |  |  |

^a^ Redundancies use the code: K (GT); R (AG); D (AGT)

^b^ Nearest neighbor Tm (degrees C)
