# Supplementary material for: Studying Seabird Diet through Genetic Analysis of Faeces: A Case Study on Macaroni Penguins (Eudyptes chrysolophus)
Source: PLoS One. 2007 Sep 5;2(9):e831. doi: 10.1371/journal.pone.0000831 (PMC1959119; doi:10.1371/journal.pone.0000831)
Supplement: Figure S1 — Collection dates for all 88 penguin faecal samples analysed during the study and sample numbers for the 39 penguin faecal samples containing prey DNA. Sample numbers correspond to clone library results in Table S3 and presence/absence results shown in Figure 1. (0.05 MB DOC) [file pone.0000831.s005.doc]

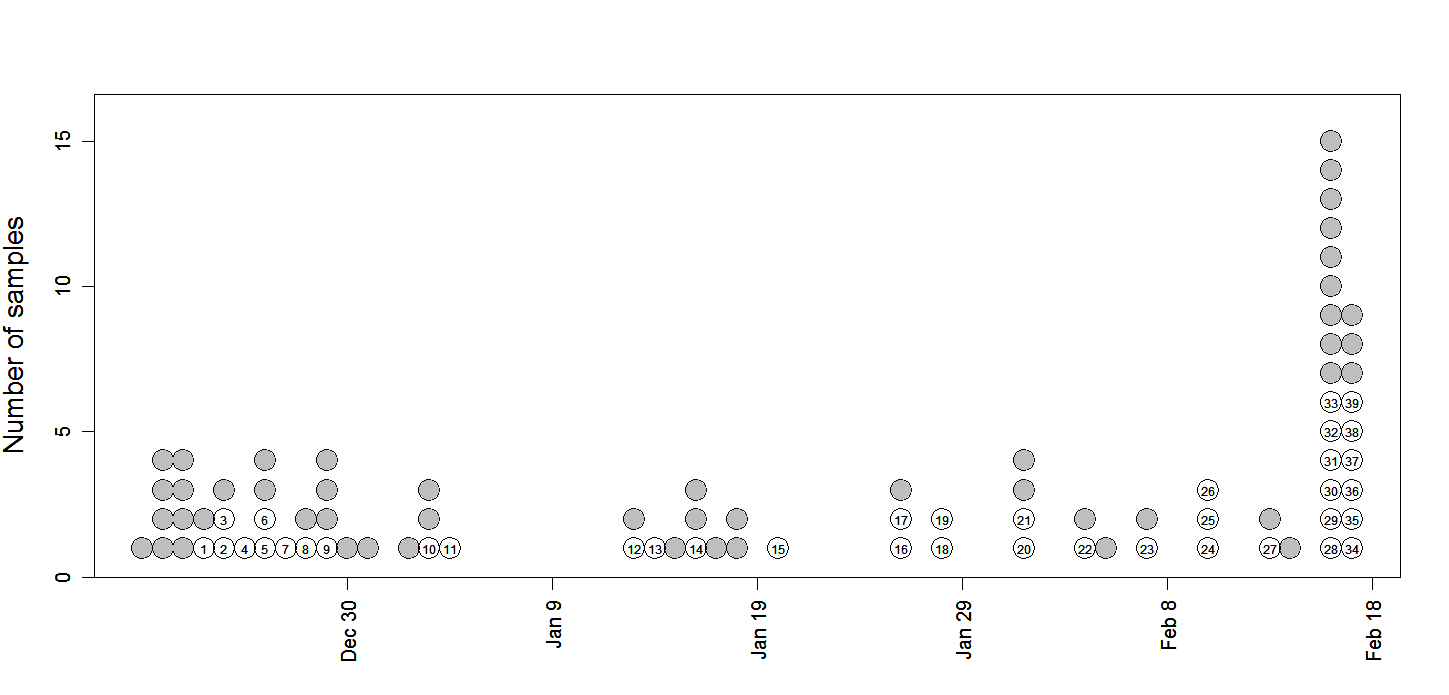


**Figure S1** Collection dates for all 88 *penguin faecal samples analysed during the study (each represented by a dot). Sample numbers are shown for the 39 samples containing detectable prey DNA; filled grey dots represent samples with no detectable prey DNA. Sample numbers correspond to clone library results in Table S2 (Sample #) and presence/absence results shown in Figure 1.*
